# Regenerative and Antioxidant Properties of Autologous Platelet-Rich Plasma Can Reserve the Aging Process of the Cornea in the Rat Model

**DOI:** 10.1155/2020/4127959

**Published:** 2020-11-23

**Authors:** Heba R. Hashem

**Affiliations:** Anatomy and Embryology Department, Faculty of Medicine, Ain Shams University, Cairo, Egypt

## Abstract

Aging is a natural progressive decline in the biological function of cells. Age-related changes in the cornea can affect its ability to refract light or repair itself. Platelet-rich plasma (PRP) has a promising role in regenerative medicine and evidenced its efficacy in multiple fields, but in corneal aging has not yet been elucidated. The present work was performed to estimate the regenerative antioxidant effect of PRP on corneal aging in rats. Rats were assigned into two main groups: (GI) adult group and (GII) aged group. The adult group was divided into GIa (adult rats), GIb (adult-saline treated), and GIc (adult-PRP treated). The aged group was divided into GIIa (aged rats) and GIIb (aged, PRP treated). PRP was administered by a single subconjunctival injection. After 10 days, histological, ultrastructural, immunohistochemical, and morphometrical investigations were carried out. Examination of the corneal sections of the aged group revealed corneal epithelial thinning, shedding of the surface epithelium with loss of desmosomal junction, and irregularity in Bowman's membrane. Disorganized widely spaced collagen bundles and neovascularization were detected in corneal stroma associated with thickening in Descemet's membrane. Ultrastructural examination revealed shrunken hyperchromatic nuclei, swollen mitochondria, and scanty cytoplasm with a strong nuclear reaction for caspase-3 immunostaining. Moreover, antioxidant/free radicals' imbalance was detected by the increase of malondialdehyde (MDA) level with a decrease of glutathione peroxidase (GPx) and superoxide dismutase (SOD) levels. In contrast, GIIb (aged, PRP treated) section examination revealed a restoration of the thickness of the corneal epithelial layer and Descemet's membrane with an amendment of collagen fiber regularity that is associated with weak nuclear reaction to caspase-3 and recovery of the balance in the redox state. These findings proved the effectiveness of PRP as a promising regenerative treatment for the age-associated changes in the cornea.

## 1. Introduction

The cornea is the transparent anterior part of the eye that covers the iris, pupil, and the anterior chamber. Its major functions are to support the tear film and surface refractivity and to transmit light through its translucent tissue to the lens and then to the retina [1]. Corneal hydration is vital to protect the corneal epithelium from injuries and preservation of corneal transparency [2].

The cornea is formed of five distinct histological layers arranged from outer to inner, respectively, corneal epithelium, Bowman's membrane, stroma, Descemet's membrane, and endothelium.

The corneal epithelium (stratified squamous nonkeratinized) is one of the most sensitive and densely innervated surface tissues in the human body. It promotes the maintenance of reflex tear production and the physiologic renewal of the corneal epithelium [3]. Corneal stroma represents almost 90% of the corneal thickness [4].

The integrity of the stroma and the normal metabolism of endothelial cells are important factors to preserve corneal transparency [2]. The transparency depends on the regular spacing and uniform diameter of the collagen bundles in the stroma [5].

Moreover, Descemet's membrane is the basement membrane of the corneal endothelium cell layer. The endothelial cell layer provides a barrier function and acts as an active water pump to keep the cornea in a constant state of dehydration [6]. People are born with a fixed number of corneal endothelial cells that gradually decreased with age [1].

Aging is a natural biological progressive process characterized by a decrease in cellular and molecular tissue functions [7]. Corneal aging produces both structural and functional changes that have a major effect on vision [8]. These changes may include keratoconus, alterations of higher order aberrations of the cornea, and a rotation of the axis of astigmatism resulting in a shift from with-the-rule to against-the-rule astigmatism [9]. Age-related alterations lead to dry eye, which in turn leads to a decrease in visual acuity, discomfort, subjacent epithelial injury, and inflammation [1].

Platelet-rich plasma (PRP) becomes an attractive way of treatment in several fields like regenerative medicine, dental and plastic surgical applications, ophthalmological surgery, trauma, and skin burns [10].

PRP is an endogenously derived therapeutic technology so it is a nontoxic and nonimmunogenic technique used to accelerate and stimulate tissue healing [11]. PRP is obtained from centrifugation for the blood sample to get cellular constitute with a platelet concentration higher than that in circulating blood [12].

PRP was found to promote tissue regeneration by enhancing cell proliferation and differentiation [13]. Platelets secrete several growth factors, including insulin-like growth factors, platelet-derived growth factor, vascular endothelial growth factor, and fibroblast growth factor [11, 14]. These growth factors promote the process of collagen formation, angiogenesis, and regeneration. Moreover, the antimicrobial ability of PRP was proved by leukocytes' presence that lowering the risk of infection [13].

The present study is conducted to assess the efficacy of autologous platelet-rich plasma on the histopathological changes that occurred in the cornea during the aging process.

## 2. Materials and Methods

### 2.1. Animals

Thirty male albino rats of Wistar strain were used in the present study. Eighteen of the rats were aged 3-6 months old and were considered as adult rats, and the rest were aged 22-26 months old and were considered as aged rats [15]. Animals were obtained from the animal house of Research Center and Bilharzial Research Unit of Faculty of Medicine, Ain Shams University.

Rats were allowed free access to water and food and were housed in a wire cage with 12 hours day and night cycle. The animals were kept in adjusted laboratory conditions (temperature 21 ± 3°C, well-ventilated wire cages). Animals were left one week for acclimatization before the start of the experiment.

### 2.2. Ethical Consideration

All the experiments and animal procedures were conducted following the national guidelines approved by the Committee of Animal Research Ethics (CARE), Faculty of Medicine, Ain Shams University, and following the NIH Guidelines for the Care and Use of Laboratory Animals 8th edition.

### 2.3. Experimental Design

In Group I, twelve adult rats were divided randomly into two groups as follows:

Ia (adult, untreated group): six adult rats were not subjected to any procedure

Ib (adult, saline-treated group): six adult rats received a single subconjunctival injection of 0.5 ml of saline then left untreated for the rest of the experiment (10 days)

Ic (adult, PRP treated group): six adult rats received a single subconjunctival injection of 0.5 ml of platelet-rich plasma (PRP) then were left untreated for the rest of the experiment (10 days) [14]

In Group II, twelve aged rats were divided randomly into two groups as follows:

IIa (aged, untreated group): six aged rats were not subjected to any procedure

IIb (aged, PRP-treated group): six aged rats received a single subconjunctival injection of 0.5 ml of platelet-rich plasma (PRP) then were left untreated for the rest of the experiment (10 days) [14]

### 2.4. Preparation of Platelet-Rich Plasma (PRP)

Blood samples were collected from the rats to be injected by their PRP. Rats were anesthetized by intraperitoneal injection of 10% chloral hydrate (350 mg/kg body weight) [16]. Venous blood was collected from the tail vein in acid citrate dextrose (ACD) tubes. The samples were centrifuged at 1480 rpm for 6 minutes at 20°C to sediment down red blood cells (RBCs). The supernatant plasma containing platelets was transferred into another sterile tube without anticoagulant. Secondly, the centrifuge was done at 4000 rpm for 15 minutes at 20°C to separate platelet-rich plasma (PRP) (lower 1/3rd) from platelet-poor plasma (PPP) (the upper 2/3rd) [17]. Samples were centrifuged by using Jouan ki22 Refrigerated Centrifuge (LAB EQUIP LTD, France) and processed at the Tissue Culture and Research Center in the Faculty of Medicine, Al-Azhar University, Egypt.

At the end of the experiment, all rats were euthanized by an intraperitoneal injection of phenobarbital (50 mg/kg body weight) [18]. Then, the eyes were enucleated. Some were fixed in 10% formal saline; the cornea was dissected and processed for light microscopy. Others were fixed in glutaraldehyde and processed for the ultrastructural study.

### 2.5. Light Microscopic Study

Specimens were fixed in 10% neutral-buffered formalin, dehydrated in graded alcohol, cleared in xylol, and embedded in paraffin. Sections of 5 *μ*m thickness were stained with.

1. Hematoxylin and Eosin (HE) staining method [19]
2. Masson trichrome staining method: for the detection of collagen fibers [19]

The slides were examined and photographed with the Lecia ICC50 camera.

### 2.6. Immunohistochemical Study

Five *μ*m thick corneal sections were obtained from paraffin blocks, and the slides were dried and deparaffinized. Slides were washed in PBS. An antigen retrieval solution was applied for 10 minutes and endogenous incubation in 3% H2O2 for 10 minutes. The sections were incubated with rabbit polyclonal anticaspase-3 (Abcam, ab4051; Cambridge, UK, dilution 1 : 100) at room temperature for 90 minutes. Sections were then washed several times with PBS then incubated with secondary antibody (catalog number ab205718, Abcam, Cambridge, UK) at room temperature for 20 minutes [20]. Negative control sections were performed with the same procedure, but the primary antibody was nonimmune rabbit serum.

### 2.7. Ultrastructural Study

Corneal specimens from sacrificed rats were obtained and fixed in glutaraldehyde and osmium tetroxide. The fixed parts were dehydrated and embedded in Epon resin. Semithin sections, 1 *μ*m thick was cut and stained with 1% toluidine blue and examined by a light microscope to choose the selected areas for proper orientation. Ultrathin sections (80-90 nm) were cut with a diamond knife and stained by Uranyl acetate and lead citrate [21].

The electron microscopic study was performed with a Jeol 1010 Transmission Electron Microscope (Japan) at the Regional Center for Mycology and Biotechnology, Al-Azhar University, Egypt.

### 2.8. Tissue Homogenate (Determination of Redox Status in the Cornea)

The tissue samples were homogenized in cold phosphate buffered saline (PBS; 10% *w*/*v*) using a tissue homogenizer. The homogenate was centrifuged at 10,000 rpm for 20 min at 4°C, and the supernatant was collected.

#### 2.8.1. Malondialdehyde (MDA) Level

It is the indicator of lipid peroxidation (Colorimetric/Fluorometric Assay Kit, Catalog # K739-100, BioVision, USA). 10 mg of the sample tissue was blended with 150 *μ*l dH2O, 3 *μ*l BHT, and 1 vol of 2 N perchloric acid, vortexing then centrifuged to remove precipitated protein for 10 min (4000 rpm).

#### 2.8.2. Glutathione Peroxidase Activity (GPx)

Glutathione peroxidase family of enzymes plays an important role in the protection from oxidative damage (Colorimetric Assay Kit, Catalog #K762-100; 100 reactions; Store kit at –20°C). 0.1 g tissue sample was homogenized then centrifuge for 15 min.

#### 2.8.3. Superoxide Dismutase (SOD)

It is a group of enzymes that catalyze the superoxide radicals to hydrogen peroxide thus providing cellular defense against reactive oxygen species (Colorimetric Assay Kit, Catalog #K335-100; Store kit at –4°C). Tissue samples were washed by PBS then centrifuged at 500 rpm for 5 min.

All tissue homogenate samples were processed at Tissue Culture and Research Center, Histology Department, Al-Azhar University.

### 2.9. Morphometric Study

Images were analyzed using computer-based software, the image analyzer Leica (Q 500 MC program, Wetzlar, Germany) at Histology Department, Faculty of Medicine, Al-Azhar University, Egypt. Six different nonoverlapping randomly selected fields obtained from different animals from the same group were used to measure:

1. The thickness of the corneal epithelium in HE sections
2. The thickness of Descemet's membrane in HE sections
3. Area of the percentage of caspase-3 positive cells in immunohistochemically stained sections

### 2.10. Statistical Analysis

The obtained data were statistically analyzed using the SPSS statistical package (IBM Corporation, New York, USA), and data are presented as mean ± standard deviation (SD). Statistical analysis was performed using one-way analysis of variance (ANOVA) followed by a Tukey's post hoc multiple comparison test. The difference was considered significant at p < 0.05 and highly statistically significant at *p* < 0.001.

## 3. Results

Light and electron microscopic examination of sections of the cornea from groups Ia, Ib, and Ic showed similar findings with no observable differences. Thus, they were represented as the adult group (I) in figures.

### 3.1. Histological Results

**Figure 1:**
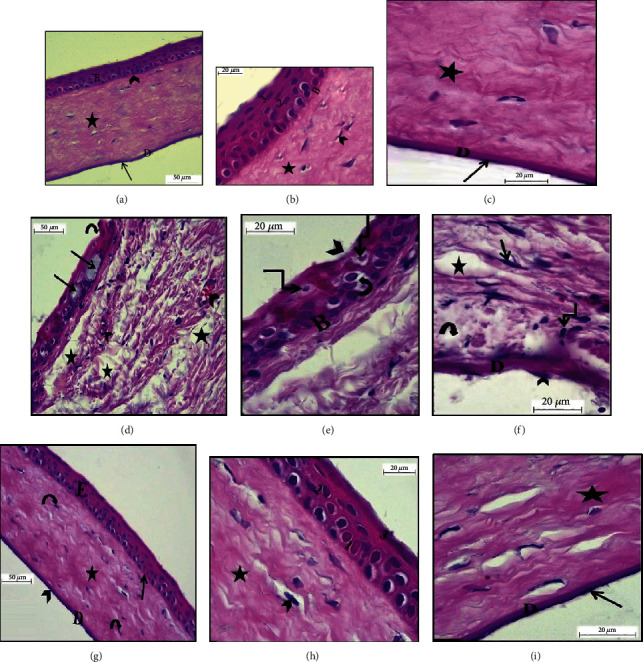
Photomicrographs of HE sections in the rat cornea showing. Group I (adult): (a) the five layers of the cornea: the corneal epithelium (E), Bowman's membrane (arrowhead), corneal stroma (asterisk), and Descemet's membrane (D). Notice the flat endothelial cells (arrow) (HE ×400); (b) Bowman's membrane (B) and layers of the corneal epithelium: basal layer (1), the intermediate layer (2), and the superficial layer (3). Notice the corneal stroma with bundles of collagen fibers (asterisk) and keratocytes in between (arrowhead) (HE ×1000); (c) collagen bundles (asterisk), Descemet's membrane layer (D), and a single layer of flat endothelial cells (arrow) on its inner surface (HE ×1000). Group IIa (aged): (d) focal discontinuity in the surface epithelium (curved arrow), pale vacuolated degenerated basal, and intermediate cells (arrow). Notice the neovascularization (arrowhead) and irregularly arranged widely spaced collagen bundles (asterisk) in the stroma (HE ×400); (e) corneal epithelial layers with focal discontinuity in the surface epithelium (arrowhead) with shrunken nuclei (angular arrow) and vacuolated cytoplasm (curved arrow). Notice the thin irregular Bowman's membrane with an area of focal disruption (B) (HE ×1000); (f) stromal inflammatory cellular infiltration (angular arrow), keratocytes (arrow), and irregularly arranged widely spaced collagen bundles (asterisk). Notice the irregular degenerated Descemet's membrane (D) with areas of separation from corneal stroma (curved arrow) and irregularly arranged endothelial cells (arrowhead) (HE ×1000). Group IIb (aged, PRP treated): (g) the cornea layers arranged in its normal order as corneal epithelium (E), Bowman's membrane (arrow), stromal collagen fibers (asterisk) with keratocytes (curved arrow), and Descemet's membrane (D) with endothelial cells (arrowhead) (HE ×400); (h) layers of the corneal epithelium: basal layer (1), the intermediate layer (2), the superficial layer (3), and the stroma collagen fibers (asterisk) with keratocytes in between (arrowhead) (HE ×1000); (i) Descemet's membrane layer (D) and a single layer of flat endothelial cells (arrow) on its inner surface. Notice collagen bundles (asterisk) (HE ×1000).

Examination of HE stained sections of the cornea obtained from the adult group showed the typical structure of the cornea; the corneal epithelium is the outer layer which was formed of stratified squamous nonkeratinized epithelium appeared with superficial flat squamous cells having flat nuclei, intermediate polygonal cells with rounded nuclei, and basal columnar cells with oval nuclei. The second layer is the Bowman's membrane which was a thin homogenous layer consisting of fibrous tissue that lies beneath the corneal epithelium (Figures 1(a) and 1(b)). The avascular corneal stroma was the third layer. It contains regularly arranged bundles of collagen fibers with spindle-shaped keratocytes in between (Figures 1(a)–1(c)). The next layer the Descemet's membrane which appeared homogenous continuous beneath the stroma and was covered by an inner single layer of flat endothelial cells which was the fifth layer (Figures 1(a) and 1(c)).

Examination of HE-stained sections of the aged group showed disfigurement of the normal corneal histological structure. The corneal epithelial layer showed focal discontinuity and denudation in the surface epithelium. The basal and intermediate layer appeared with pale vacuolated cytoplasm and deeply stained nuclei. Bowman's membrane appeared very thin, irregular with areas of disruption (Figures 1(d) and 1(e)). Stromal inflammatory cellular infiltration and neovascularization were observed accompanied by disruption, irregularly arranged, and widely spacing collagen bundles (Figures 1(d) and 1(f)). Descemet's membrane appeared irregular and degenerated with areas of separation from the corneal stroma. That was accompanied by an irregular arrangement or loss of some endothelial cells (Figure 1(f)).

Examination of HE stained sections of aged, PRP-treated group revealed an apparently normal corneal epithelium in comparison to the adult group with regular intact Bowman's membrane (Figure 1(g)). Cytoplasmic vacuolation was noticed in few epithelial cells. Corneal stroma had regularly arranged collagen fibers with few spaces and keratocytes in between. Endothelial cells and Descemet's membrane were apparently normal (Figures 1(h) and 1(i)).

### 3.2. Masson Trichrome Staining Results

**Figure 2:**
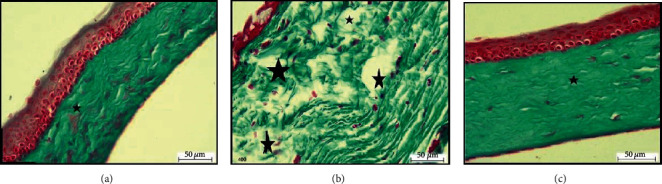
Photomicrographs of Masson trichrome sections in the rat cornea. (a) Group I (adult) section showing regularly arranged collagen fibers in the corneal stroma (asterisk) (Masson Trichrome ×400). (b) Group IIa (aged) section showing disorganized widely separated collagen fibers in the corneal stroma (asterisk) (Masson Trichrome ×400). (c) Group IIb (aged, PRP treated) section showing regularly arranged collagen fibers in the corneal stroma (asterisk) (Masson Trichrome ×400).

Examination of Masson trichrome stained sections of the adult group revealed regularly arranged collagen bundles in the stroma (Figure 2(a)). However, Masson's trichrome stained cornea sections of the aged group showed disorganized, interrupted, and widely separated stromal collagen fibers (Figure 2(b)). On the other hand, Masson's trichrome stained cornea sections from aged, PRP-treated group showed regularly arranged collagen fibers (Figure 2(c)).

### 3.3. Immunohistochemical Results

**Figure 3:**
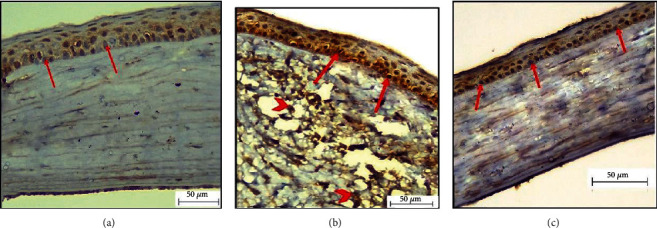
Photomicrographs of caspase-3 sections in the rat cornea. (a) Group I (adult) section showing weak immune reaction in the corneal epithelium nuclei (arrow) (caspase-3 ×400). (b) Group IIa (aged) showing a strong immune reaction in nuclei of the corneal epithelium (arrow) and some keratocytes (arrowhead) (caspase-3 ×400). (c) Group IIb (aged, PRP treated) section showing weak immune reaction in the corneal epithelium nuclei (arrow) (caspase-3 ×400).

Immunohistochemically, caspase-3 stained cornea sections from the adult group showed a weak immune reaction in the corneal epithelium nuclei (Figure 3(a)). In the aged group, immunohistochemically stained sections of caspase-3 showed a strong immune reaction in nuclei of corneal epithelium and some keratocytes (Figure 3(b)), but in the aged, PRP-treated group, immunohistochemically stained sections showed a weak immune reaction in the corneal epithelium (Figure 3(c)).

### 3.4. Semithin Sections

**Figure 4:**
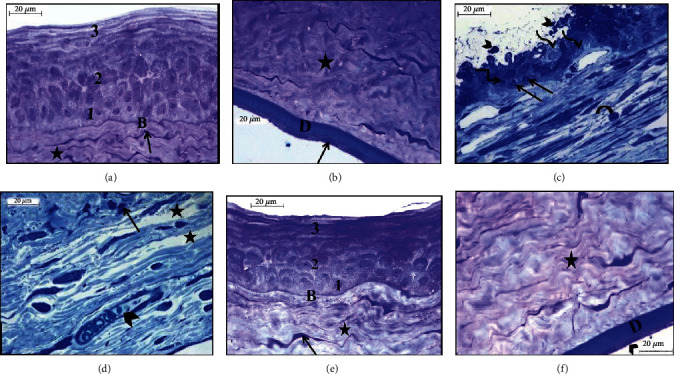
Photomicrographs of semithin sections in the rat cornea showing group I (adult): (a) layers of corneal epithelium: basal layer (1), the intermediate layer (2), and the superficial layer (3) that resting on regular Bowman's membrane (B). The collagen bundles of avascular stroma (asterisk) with keratocytes in between (arrow) (Toluidine blue ×1000); (b) thick homogenous Descemet's membrane layer (D) with a layer of flat endothelial cells resting on its inner surface (arrow) and stromal collagen fibers (asterisk) (Toluidine blue ×1000). Group IIa (aged): (c) detached epithelial cells (arrowhead), pale vacuolated cytoplasm of basal, and intermediate cell layers (wavy arrow) with deeply stained nuclei that some of them are pyknotic (angular arrow) and others are shrunken (arrow). Notice the eosinophils (curved arrow) in the corneal stroma (Toluidine blue ×1000); (d) irregular arranged widely spaced collagen bundles (asterisk), the neovascularization (arrowhead), and shrunken nuclei of keratocytes (arrow) (Toluidine blue ×1000). Group IIb (aged, PRP treated): (e) layers of corneal epithelium arranged as an inner basal layer (1), the intermediate layer (2), and outer superficial layer (3). Notice the regular Bowman's membrane (B) and collagen bundles of avascular stroma (asterisk) with keratocytes (arrow) (Toluidine blue ×1000); (f) corneal stroma collagen fibers (asterisk) and homogenous Descemet's membrane (D) with a single layer of flat endothelial cells (arrowhead) (Toluidine blue ×1000).

Examination of the semithin sections of rat cornea from the adult group showed the three layers of corneal epithelium arranged as the inner basal columnar cells, intermediate multilayered polygonal cells, and the outer squamous layer. Bowman's membrane appeared as a thin regular membrane that basal cells resting on (Figure 4(a)). Avascular stroma appeared with regularly arranged collagen lamella with spindle-shaped keratocytes in between (Figures 4(a) and 4(b)). Descemet's membrane layer appeared thick and homogenous with an inner single layer of flat endothelial cells resting on (Figure 4(b)).

However, the examination of semithin cornea sections of the aged group revealed that disorganization and sloughing of cells in the surface epithelium. Basal and intermediate layers showed pale vacuolated cytoplasm with deeply stained nuclei that some of them were pyknotic and others were shrunken. Bowman's membrane appeared irregular, thin, and difficult to be detected (Figure 4(c)). Irregular collagen fibers observed in corneal stroma accompanied by wide spacing, nuclear changes in keratocytes, and neovascularization (Figure 4(d)).

In the aged, PRP-treated group, semithin cornea sections showed apparently normal cornea histology in comparison to the control group. The corneal epithelium layer basal cells, the intermediate layer, and the superficial squamous layer appeared like control (Figure 4(e)). Stromal collagen fibers showed regularly arranged with keratocytes in-between. Neovascularization and inflammatory infiltration cannot be detected (Figures 4(e) and 4(f)). Descemet's membrane appeared homogenous and regular with endothelial cells arranged regularly on it (Figure 4(f)).

### 3.5. Ultrathin Sections

**Figure 5:**
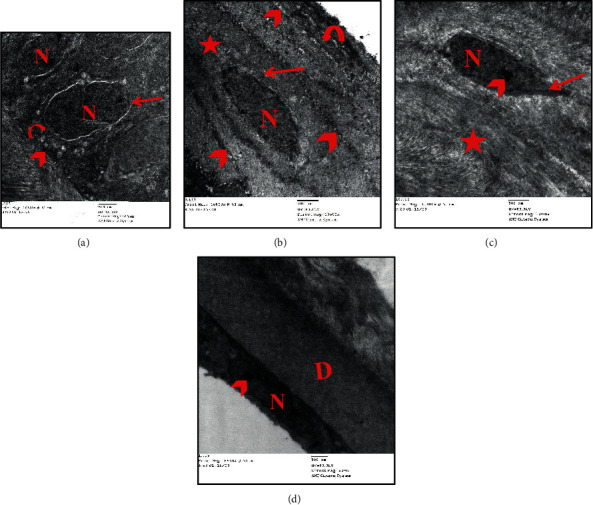
Photomicrographs of transmission electron micrograph sections in the rat cornea of group I (adult) showing (a) a single layer of basal columnar cells of the corneal epithelium with euchromatic nucleus (N), regular nuclear membrane (arrow), and mitochondria (curved arrow) resting on Bowman's membrane (arrowhead) (Uranyl acetate and lead citrate ×10000); (b) the polygonal cells of the intermediate layer of corneal epithelium with oval nuclei (N), mitochondria (arrow), and the squamous cells of the superficial layer with a flat nucleus (curved arrow), numerous electron-dense desmosomes (arrowhead), and narrow intercellular spaces (asterisk) (Uranyl acetate and lead citrate ×10000); (c) avascular corneal stroma with regularly arranged collagen fibers (asterisk) and spindle-shaped keratocytes with an oval nucleus (N), prominent nucleus (arrowhead), and scanty cytoplasm (arrow) (Uranyl acetate and lead citrate ×10000); (d) Descemet's membrane as an acellular homogenous electron-dense layer (D) and a single layer of endothelial cells with a flat electron-dense nucleus (N), with a prominent nucleolus (arrowhead) (Uranyl acetate and lead citrate ×8000).

Electron microscopic examination of the rat cornea from the adult group revealed histological structure. The corneal epithelium appeared to be formed of a basal layer of columnar cells with a euchromatic nucleus that was resting on the basement membrane (Bowman's membrane) (Figure 5(a)). The intermediate cell layer and the superficial layer appeared attached with numerous electron-dense desmosomes with narrow intercellular spaces. These cells showed few cytoplasmic organelles (Figure 5(b)). The corneal stroma was formed of regularly arranged collagen fibers with keratocytes in-between. The keratocytes were spindle-shaped cells with an oval nucleus, prominent nucleolus, and scanty cytoplasm (Figure 5(c)). Descemet's membrane appeared as an acellular thick homogenous electron-dense layer. A single layer of endothelial cells with flat electron-dense nuclei and scanty cytoplasm was resting on the Descemet's membrane (Figure 5(d)).

**Figure 6:**
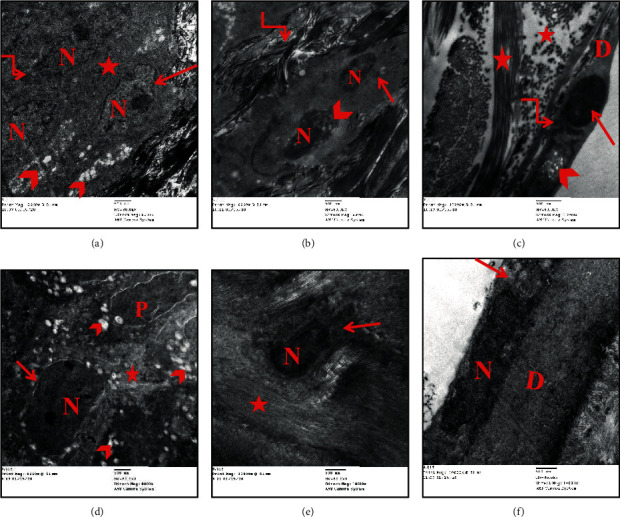
Photomicrographs of transmission electron micrograph sections in the rat cornea showing group IIa (aged): (a) corneal epithelial cells (basal and intermediate cells) with irregular nuclear membrane (arrow), shrunken hyperchromatic nucleus (N), and swollen mitochondria (angular arrow). Notice the multiple cytoplasmic vacuolations (arrowhead) and widening of the intercellular spaces with loss of desmosomal junctions (asterisk) (Uranyl acetate and lead citrate ×8000); (b) corneal stroma with irregularly arranged collagen bundles (angular arrow) and degenerated keratocytes with shrunken nuclei (N), shrunken mitochondria (arrow), and cytoplasmic vacuolation (arrowhead) (Uranyl acetate and lead citrate ×8000); (c) collagen fibers with different directions (asterisk), Descemet's membrane (D), and endothelial cell with degenerated electron-dense nucleus (arrow), shrunken mitochondria (angular arrow), and cytoplasmic vacuolation (arrowhead) (Uranyl acetate and lead citrate ×12000). Group IIb (aged, PRP treated): (d) euchromatic nucleus of the basal columnar cell (N) with regular nuclear membrane (arrow) and euchromatic nucleus of the polygonal of the intermediate layer (P). Notice the cytoplasmic vacuolation (arrowhead) with a slight widening of intercellular spaces (asterisk) (Uranyl acetate and lead citrate ×8000); (e) regularly arranged collagen fibers of the corneal stroma (asterisk) and spindle-shaped keratocytes with an oval nucleus (N) and scanty cytoplasm (arrow) (Uranyl acetate and lead citrate ×10000); (f) regular homogenous Descemet's membrane (D) and electron-dense elongated nucleus of endothelial cells (N) with small vacuoles (arrow) (Uranyl acetate and lead citrate ×10000).

Ultrastructural examination of the aged group corneal sections revealed marked disfigurement of normal corneal ultrastructural. The corneal epithelium showed multiple cytoplasmic vacuolations, focal separation, and widening of the intercellular spaces with loss of desmosomal junctions. Corneal epithelial cells showed disfigurement in their shapes. They appeared with irregular nuclear membrane, shrunken hyperchromatic nucleus, and swollen mitochondria (Figure 6(a)). Corneal stroma showed irregularly arranged collagen fibers with wide spaces. Degenerated keratocytes appeared with shrunken nuclei, degenerated mitochondria, and cytoplasmic vacuolation (Figure 6(b)). Endothelial cells appeared with a degenerated electron-dense nucleus and shrunken mitochondria with cytoplasmic vacuolation (Figure 6(c)).

Moreover, the ultrastructure examination of the corneal section of the aged, PRP-treated group revealed apparently like the control group. However, the corneal epithelium showed cytoplasmic vacuolation with a slight widening of intercellular spaces (Figure 6(d)). The stroma appeared almost normal with narrow spaces, regularly arranged collagen fibers, and keratocytes with a euchromatic nucleus and scanty cytoplasm (Figure 6(e)). Descemet's membrane appeared thick homogenous with normal endothelial cells with an electron-dense elongated nucleus. Few small vacuoles were observed in a few endothelial cells (Figure 6(f)).

### 3.6. Morphometric and Biochemical Results

Morphometric and biochemical results from groups Ia, Ib, and Ic showed similar findings with no statistical differences.

#### 3.6.1. The Thickness of the Corneal Epithelium

**Figure 7:**
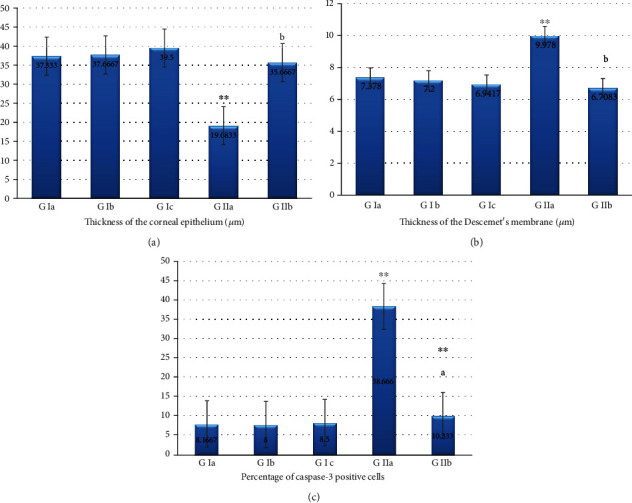
Effect of PRP treatment on the structure of the cornea in the studied age groups of animals. All data represented as mean ± SEM. Data were analyzed by one-way ANOVA using Tukey's post hoc test. (a) Histogram showing the thickness of the corneal epithelium (^∗∗^, highly statistically significant versus adult group) (b, statistically (*p* < 0.05) versus aged group (*p* < 0.001)). (b) Histogram showing the thickness of the Descemet's membrane (^∗∗^, highly statistically significant versus adult group) (b, statistically (*p* < 0.05) versus aged group (*p* < 0.001)). (c) Histogram showing percentage of caspase-3 positive cells (^∗∗^, highly statistically significant versus adult group) (a, statistically (*p* < 0.05) versus aged group (*p* < 0.001)).

In group Ia (adult rats), the corneal epithelium thickness was 37.33 ± 1.966 *μ*m (mean ± SD). In group IIa (aged rats), it was 19.083 ± 2.835 *μ*m. It showed a highly statistically significant decrease when compared to group Ia (*p* < 0.001). In group IIb (aged, PRP-treated rats), it was 35.667 ± 3.326 *μ*m where it showed a highly statistically significant increase when compared to group IIa (*p* < 0.05) (Figure 7(a)).

#### 3.6.2. The Thickness of Descemet's Membrane

In group Ia (adult rats), the Descemet's membrane thickness was 7.378 ± 0.819 *μ*m (mean ± SD). In group IIa (aged rats), it was 9.978 ± 0.335 *μ*m. It showed a highly statistically significant increase when compared to group Ia (*p* < 0.001). In group IIb (aged, PRP-treated rats), it was 6.708 ± 0.548 *μ*m where it showed a highly statistically significant decrease when compared to group IIa (*p* < 0.05) (Figure 7(b)).

#### 3.6.3. Area of the Percentage of Caspase-3 Positive Cells

In group Ia (adult rats), the percentage of caspase-3 positive cells was 8.166 ± 1.169 (mean ± SD). In group IIa (aged rats), it was 38.66 ± 7.146. It showed a highly statistically significant increase when compared to group Ia (*p* < 0.001). In group IIb (aged, PRP-treated rats), it was 10.33 ± 1.639 *μ*m where it showed a highly statistically significant decrease when compared to group IIa (*p* < 0.05) (Figure 7(c)).

#### 3.6.4. Malondialdehyde (MDA) Level

**Figure 8:**
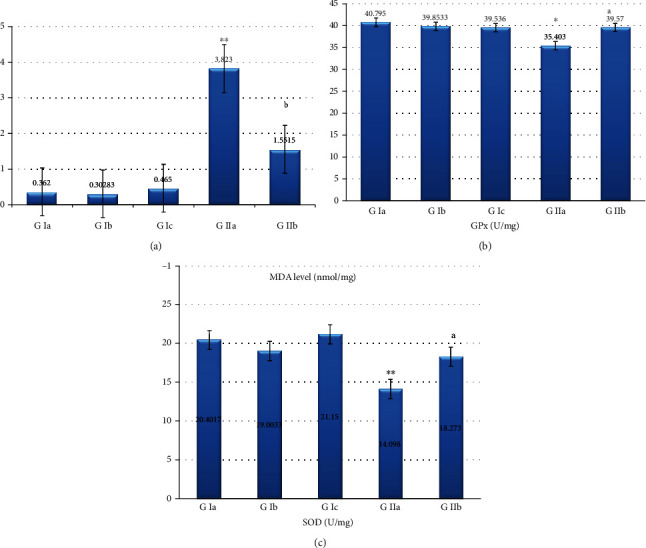
Effect of PRP treatment on the level of oxidative stress in the corneal tissue of the studied groups of animals. All data represented as mean ± SEM. Data were analyzed by one-way ANOVA using Tukey's post hoc test. (a) Histogram showing the MDA level (^∗∗^, highly statistically significant versus adult group) (b, statistically (*p* < 0.05) versus aged group (*p* < 0.001)). (b) Histogram showing the GPx level (^∗^, statistically (*p* < 0.05) versus adult group) (a, statistically (*p* < 0.05) versus aged group). (c) Histogram showing SOD level (^∗∗^, highly statistically significant versus adult group) (a, statistically (*p* < 0.05) versus aged group (*p* < 0.001)).

In group Ia (adult rats), the MDA level was 0.4453 ± 0.175 nmol/mg (mean ± SD). In group IIa (aged rats), it was 3.323 ± 0.717 nmol/mg. It showed a highly statistically significant increase when compared to group Ia (*p* < 0.001). In group IIb (aged, PRP-treated rats), it was 1.0015 ± 0.553 nmol/mg where it showed a statistically significant decrease when compared to group IIa (*p* < 0.05) (Figure 8(a)).

#### 3.6.5. Glutathione Peroxidase Activity (GPx)

In group Ia (adult rats), GPx level was 40.795 ± 2.06 U/mg (mean ± SD). In group IIa (aged rats), it was 35.403 ± 2.097 U/mg. It showed a statistically significant decrease when compared to group Ia (*p* < 0.05). In group IIb (aged, PRP-treated rats), it was 39.57 ± 2.04 U/mg where it showed a statistically significant increase when compared to group IIa (*p* < 0.05) (Figure 8(b)).

#### 3.6.6. Superoxide Dismutase (SOD)

In group Ia (adult rats), the SOD level was 20.4017 ± 1.8209 U/mg (mean ± SD). In group IIa (aged rats), it was 14.098 ± 1.997 U/mg. It showed a highly statistically significant decrease when compared to group Ia (*p* < 0.05). In group IIb (aged, PRP-treated rats), it was 18.273 ± 1.566 U/mg where it showed a statistically significant increase when compared to group IIa (*p* < 0.05) (Figure 8(c)).

## 4. Discussion

The cornea is the primary refracting component of the visual system, and it is a part of the anterior segment of the eye that acts like a shield to keeps clear vision and protects against loss of fluids and pathogen invasion [22].

Aging is an intrinsic complex process. It is accompanied by a progressive loss of function and an increased mortality rate [23]. Aging is accompanied by the free radicals induced alterations of properties of the cell membrane including decreased fluidity, alteration in the electron transport chain complexes activities, and mitochondrial failure [24].

Oxidative stress plays an important role in the development and progression of the aging process which occurred due to an imbalance between free radicals and the antioxidants system [23, 24]. In the eye, oxidative damage can result xin molecular changes that contribute to the development of age-related diseases such as cataracts and glaucoma [25].

Reactive oxygen species (ROS) may be a by-product of cellular aerobic metabolism or signaling molecules of stress in response to cellular damage [25, 26]. The production of ROS has to be balanced with reducing agents and antioxidant enzymes which protect the tissues against oxidative stress. However, when their production exceeds the antioxidant capacity, they induce damage to cell components such as DNA, proteins, and lipids [25].

The oxidative stress intensity may be estimated by the measurement of malondialdehyde (MDA) [26]. MDA is the main product of free radicals and an indicator of lipid peroxidation which is considered as a biomarker of aging [27, 28]. In the present study, there is a highly statistically significant increase in the MDA level in the aged rat group in comparison to the adult rat group.

On the contrary, the main reducing system in the eye is the glutathione system. Glutathione peroxidase (GPx) is a family of enzymes that plays an important role in the protection from oxidative damage as it leads to the oxidation of glutathione (GSH) to oxidized glutathione (GSSG) [25, 29]. A statistically significant decrease in GPx level was detected in the aged rat group in comparison to the adult rat group.

Additionally, superoxide dismutase (SOD) is an antioxidant enzyme that plays an important protective role against ROS and is considered as the first line of antioxidant defense [25, 26]. Free radicals are converted by SOD into hydrogen peroxide which can form a highly reactive hydroxyl ion that reacts with phospholipids in cell membranes and proteins [30]. A highly statistically significant decrease in the SOD level was detected in the aged rat group in comparison to the adult rat group.

In the current study, the histological and ultrastructural examination of the corneal sections of the aged rat's group revealed marked structural changes that affected all the corneal layers.

Focal discontinuity with sloughing of the surface epithelium was detected accompanied by a highly statistically significant decrease in the corneal epithelium of the aged group in comparison to the adult group. Thinning of the corneal epithelium might be due to a decrease in the capacity of corneal epithelial cells to proliferate [15]. Aseta et al. [31] reported that the epithelial thinning and cell loss detected in case of eye closure may be secondary to hypoxia.

Elwan and Kassab [18] demonstrate corneal epithelial thinning in the case of capecitabine-induced corneal toxicity that might be due to cellular degeneration and slow rate of cellular regeneration and proliferation.

Faragher et al. [8] mentioned that aging is associated with a reduction in the epithelial barrier function and increased epithelial permeability with changes in the distribution of integrin subunits.

Meanwhile, the epithelial cells of the aged cornea had shrunken hyperchromatic nuclei, irregular nuclear membrane, and swollen mitochondria with pale vacuolated cytoplasm. Elwan and Kassab [18] demonstrated pyknotic nuclei in the corneal epithelial cells in the case of corneal toxicity. These findings were interpreted as apoptosis by the authors.

Aseta et al. [31] explained the cytoplasmic vacuolation observed in case of eye closure as a reflection for a form of programmed cell death (type III cytoplasmic cell death) that is triggered by insulin-like growth factor or tumor necrosis factor.

Moreover, Bowman's membrane in the aged cornea was very thin and irregular that associated with widening in intercellular spaces and loss of desmosomal junctions. Ebrahim et al. [17] mentioned similar findings in the case of corneal alkali burns in rats due to corneal epithelial cells desquamation and necrosis. Also, Aseta et al. [31] detected these changes in case of eye closure and clarified that any changes occurred in the barrier function of the corneal epithelium will affect the integrity of the cornea that leads to an increased risk of infections, corneal diseases, and corneal damage.

In the current study, corneal stroma in the HE sections of the aged cornea showed various structural changes detected in the form of disarrangement and spacing in the collagen bundles with the appearance of neovascularization and inflammatory cellular infiltration. Halawa [15] observed in the stroma, wide separation of the corneal lamellae with invasion by blood vessels. She suggested that this will result in cornea edema and affect corneal transparency. Daxer et al. [32] reported that the spacing resulted from glycation-induced cross-linking and reduction of the molecular tilting angle within collagen fibrils.

The examination of the Masson trichrome stained section confirmed the stromal finding of HE sections. Corneal opacity and dry eye are a common complaint in old age caused by associated corneal edema due to widely spaced collagen lamella and reduction of sodium and potassium-dependent ATPase enzyme levels [2].

Elwan and Kassab [18] reported that stromal inflammatory cellular infiltration may be due to the release of interleukin 1*β* (IL1*β*) from the injured corneal epithelial cells in the rat model of capecitabine-induced injury.

A healthy functioning cornea is avascular. The circulating vascular endothelial growth factor (VEGF) receptors play an important role in corneal avascularity through binding with the free VEGF thereby making it unavailable for binding to receptors on cell membranes [33].

Faruk et al. [34] detected the neovascularization in histological and ultrastructural sections of a rat model of induced corneal alkali burn. Berthaut et al. [33] mentioned that the expression of VEGF receptors decreased with age in humans. On the other hand, Tendler et al. [35] revealed that neovascularization could be secondary to associated corneal inflammation and edema.

Corneal neovascularization is a major, sight-threatening complication of some ocular disorders. It results from an imbalance between angiogenic and antiangiogenic factors and is characterized by overproliferation, migration, and capillary tube formation by endothelial cells [36].

In the current study, keratocytes in aged cornea sections appeared with shrunken nuclei, shrunken mitochondria, and vacuolated cytoplasm. Keratocytes are considered as corneal fibroblasts that are involved in the process of inflammation [37]. Wang et al. [38] revealed the disarrangement of the collagen fibers due to keratocyte apoptosis.

This theory was supported by the results of caspase-3 immune-histochemical stained section examination which revealed a highly statistically significant increase in caspase-3 positive cells in corneal epithelium and keratocytes.

Rhim et al. [39] mentioned a higher level of caveolin-1 was detected in the corneal epithelium of elderly patients than the young one that indicated an increased average of cell damage. Also, the increase of TGF-b in aged cornea reflecting an increase of apoptotic death [7].

On the other hand, a highly statistically significant increase in Descemet's membrane thickness was detected in the aged group in comparison to the adult group with areas of separation between the membrane and the corneal stroma. Swollen endothelial cells with a degenerated electron-dense nucleus, shrunken mitochondria, and cytoplasmic vacuolation were observed.

Descemet's membrane (DM) is the basement membrane of the corneal endothelium composed predominately of type VIII collagen. It plays a crucial role in endothelial cell differentiation and proliferation and structural integrity [40]. The corneal endothelial layer has an important function to maintain corneal transparency. Loss of endothelial cells leads to irreversible corneal edema [41].

Jun et al. [42] detected an age-dependent focal thickening in Descemet's membrane in mice. Roh et al. [43] demonstrated the thickening of Descemet's membrane with an electron microscopy examination. Mousa et al. [2] and Farid et al. [44] explained the changes in these layers resulted from the failure of the cross-linking of collagen with the degeneration of endothelial cells. Jun et al. [42] revealed that there is an aging-associated accumulation of the posterior, nonbanded portion to this layer.

PRP is an autologous serum that is known to be safe, effective, and powerful to repair tissue damage depending on its high concentration of platelets that contain a wide variety of growth factors [45, 11]. PRP application has promising results in acute as well as chronic injuries [46]. Recently, PRP is used in many medical fields such as regenerative medicine, dental implants and prostheses, maxillofacial surgery, plastic, orthopedics, treatment diabetic ulcer, and dry eye [13, 47].

To the best of our knowledge, it has not been reported for the usage of PRP on spontaneous age-associated corneal changes.

In the current work, the examination of histological and ultrastructural corneal sections of the aged, PRP-treated group revealed a normal histological structure comparable to the adult group. It showed intact Bowman's membrane and arranged corneal epithelium in its three layers. Moreover, well-organized collagen lamella in the stroma with keratocytes situated in-between and continuous regular Descemet's membrane with regularly situated endothelial cells. However, ultrastructural examination revealed vacuolated cytoplasm of epithelial cells with wide intercellular spaces. A highly statistically significant increase and decrease in corneal epithelium thickness and Descemet's membrane thickness were detected, respectively.

Sharaf Eldin et al. [14] and Charalambidou et al. [48] detected similar findings after using PRP in a rat model of corneal alkali burn, and Tanidir et al. [49] reported the same in a rabbit model of corneal epithelium wound healing.

They explained that due to a high concentration of platelets that contained growth factors attached to the corneal surface and accelerate tissue repair mechanism. Zheng et al. [16] and Mansy et al. [50] mentioned that PRP played a dominant role in cell migration and proliferation.

Moreover, the examination of sections revealed a reduction in inflammatory cellular infiltration, vacuolation, and neovascularization. Çirci et al. [51] and Martini et al. [52] explained that PRP has a strong anti-inflammatory and antibacterial effect that reduce cellular inflammation markers. Lee et al. [45] reported that using PRP in treatment reduces inflammation and pain and enhances the healing process.

In the current study, the examination of caspase-3 immunohistochemical stained sections shows a weak immune reaction and a highly statistically significant decrease in the aged, PRP-treated group in comparison to the aged group. Rah et al. [53] stated that PRP acts as an apoptosis regulatory messenger as it reduces the expression of ASK-1, a typical member of the mitogen-activated protein kinase kinase kinase (MAPKKK) family and the critical component in ROS-induced apoptosis.

Additionally, a statistically significant decrease in the MDA level was accompanied by a statistically significant increase in GPx and SOD levels. Rah et al. [53] and Chen et al. [54] mentioned that PRP had an antioxidant effect due to its content of numerous growth factors. These growth factors resulted in a decrease in oxidative stress and a reduction in reactive oxygen species. Also, these growth factors enhance the healing processes such as cell proliferation, differentiation, and regeneration of damaged tissues through increase nutrient influx and blood supply [55, 56].

## 5. Conclusion

The findings of the current study can highlight evidence about the efficient regenerative role of single subconjunctival injection of autologous PRP in ameliorating the age-related changes in rat's cornea. That can be concluded through amelioration of the redox state and restoration of the structure of a normal healthy cornea.

## Data Availability

The data used to support the findings of this study are available upon request.
